# Effects of ATRA combined with citrus and ginger-derived compounds in human SCC xenografts

**DOI:** 10.1186/1471-2407-10-394

**Published:** 2010-07-26

**Authors:** Heather E Kleiner-Hancock, Runhua Shi, Angela Remeika, Delira Robbins, Misty Prince, Jennifer N Gill, Zanobia Syed, Patrick Adegboyega, J Michael Mathis, John L Clifford

**Affiliations:** 1 Department of Pharmacology, Toxicology & Neuroscience, Louisiana State University Health Sciences Center-Shreveport, 1501 Kings Hwy, Shreveport, Louisiana, 71103 USA; 2 Department of Medicine, Louisiana State University Health Sciences Center-Shreveport, 1501 Kings Hwy, Shreveport, Louisiana, 71103 USA; 3 Department of Biochemistry and Molecular Biology, Louisiana State University Health Sciences Center-Shreveport, 1501 Kings Hwy, Shreveport, Louisiana, 71103 USA; 4 Department of Pathology, Louisiana State University Health Sciences Center-Shreveport, 1501 Kings Hwy, Shreveport, Louisiana, 71103 USA; 5 Department of Cellular Biology & Anatomy, Louisiana State University Health Sciences Center-Shreveport, 1501 Kings Hwy, Shreveport, Louisiana, 71103 USA; 6 Center for Experimental Cancer Therapeutics, Cancer Prevention & Control Group, Feist-Weiller Cancer Center, 1501 Kings Hwy, Shreveport, LA, 71103 USA

## Abstract

**Background:**

NF-κB is a survival signaling transcription factor complex involved in the malignant phenotype of many cancers, including squamous cell carcinomas (SCC). The citrus coumarin, auraptene (AUR), and the ethno-medicinal ginger (Alpinia galanga) phenylpropanoid, 1'-acetoxychavicol acetate (ACA), were previously shown to suppress 12-*O*-tetradecanoylphorbol-13-acetate (TPA) induced mouse skin tumor promotion. The goal of the present study was to determine whether AUR and ACA are effective either alone or in combination with all-*trans *retinoic acid (ATRA) for suppressing SCC tumor growth.

**Methods:**

We first determined the effects of orally administered ACA (100 mg/kg bw) and AUR (200 mg/kg bw) on lipopolysaccharide (LPS)-induced NF-κB activation in NF-κB-RE-luc (Oslo) luciferase reporter mice. Dietary administration of AUR and ACA ± ATRA was next evaluated in a xenograft mouse model. Female SCID/bg mice were fed diets containing the experimental compounds, injected with 1 × 10^6 ^SRB12-p9 cells s.c., palpated and weighed twice a week for 28 days following injection.

**Results:**

Both ACA and AUR suppressed LPS-induced NF-κB activation in the report mice. In the xenograft model, AUR (1000 ppm) and ACA (500 ppm) modestly suppressed tumor volume. However, in combination with ATRA at 5, 10, and 30 ppm, ACA 500 ppm significantly inhibited tumor volume by 56%, 62%, and 98%, respectively. The effect of ATRA alone was 37%, 33%, and 93% inhibition, respectively. AUR 1000 ppm and ATRA 10 ppm were not very effective when administered alone, but when combined, strongly suppressed tumor volume by 84%.

**Conclusions:**

Citrus AUR may synergize the tumor suppressive effects of ATRA, while ACA may prolong the inhibitory effects of ATRA. Further studies will be necessary to determine whether these combinations may be useful in the control of human SCC.

## Background

Non-melanoma skin cancer (NMSC) is the most common cancer in the U.S., with over a million new cases of the two most common forms, squamous cell carcinoma (SCC) and basal cell carcinoma (BCC), anticipated annually [1]. The more clinically aggressive form is SCC of the skin [2], which has been increasing in incidence since the 1960 s at annual rates from 4% to as much as 10% in recent years. Better control of advanced skin SCC is clearly necessary, and will be helped by improving our understanding of the molecular basis for skin carcinogenesis and of the action of chemopreventive drugs.

Nuclear factor κB (NF-κB) is activated during inflammation and carcinogenesis [3]. Lipopolysaccharide (LPS), interleukin-1β, hydrogen peroxide, and tumor necrosis factor activate NF-κB signaling through phosphorylation of its inhibitor IκBα. This leads to degradation of IκBα, phosphorylation of the p65 NF-κB subunit, and translocation of p65, along with the p50 subunit, to the nucleus. There they form a DNA binding complex and activate transcription of specific genes involved in proliferation (cyclin D1, c-myc, COX-2), angiogenesis (vascular endothelial growth factor-VEGF), antiapoptosis (survivin, TRAF1, IAP1, BclXL, FLIP) and invasion (matrix metalloproteinase 9, ICAM-1) [4].

1'-acetoxychavicol acetate (ACA) is a natural component of traditional Thai condiments, present in the seeds (15000 ppm) and rhizomes (27300 ppm) of the ethnomedicinal plant *Languas galanga *Stuntz (Zingiberaceae) [5]. In the skin model, pre-treatment of mice with ACA prior to TPA treatment in 7,12-dimethylbenz[*a*]anthracene (DMBA)-initiated mice suppressed skin tumor promotion [6]. Recently it was found that ACA blocked TNF-α induced activation of NF-κB indirectly through IκB [7]. In this regard, ACA inhibited TNF-α-induced phosphorylation and degradation of IκBα, suggesting that it inhibits the IκBα kinase, IKK. Auraptene (AUR) is a geranyloxy-coumarin obtained from citrus fruits [8], the aromatic plant Zanthoxylum schinifolium [9] and the fruits of Paliurus ramosissimus [10]. AUR is also known to suppress skin tumor promotion [8], in addition to its many other chemopreventive effects. AUR suppresses superoxide generation indirectly, most likely through inhibition of the multi-component NADPH oxidase system [8]. AUR, possessing anti-inflammatory activities [11] also suppresses ulcerative colitis and ulcerative colitis induced colon cancer [12,13]. Consistent with these observations, AUR also activated PPARγ in adipocytes, which led to the increased production of adiponection, which is known to suppress the inflammatory process [14]. These varied mechanisms and protective effects in rodent models of cancer, combined with the low risk of toxicity [15], suggest that AUR might be a good candidate for skin SCC chemoprevention.

Retinoids are a class of chemical compounds, which include active metabolites of vitamin A (retinol), as well as a diverse array of synthetic derivatives. Retinoids have been shown to modulate multiple cellular processes, including proliferation, differentiation, homeostasis, malignant transformation and apoptosis [16-18]. ATRA has long been known as one of the most effective suppressors of tumor formation in the 2-stage mouse skin chemical carcinogenesis model [19,20]. We have recently used this model to further explore the mechanism of ATRA action and have demonstrated that ATRA targets the B-Raf/Mek/Erk signaling pathway and this activity coincides with its chemopreventive activity [21]. We have also shown recently that ATRA can suppress Stat3 signaling in skin carcinogenesis, and that Stat3 activity lies downstream of the B-Raf/Mek/Erk pathway [22]. It is interesting to note that many downstream signaling effectors involved in malignancy are regulated by both Stat3 and NF-κB

**We hypothesized that **suppression of both B-Raf/Mek/Erk/Stat3 and NF-κB signaling through combined administration of ATRA and ACA/AUR, may exert a stronger anti-tumor effect than either agent alone.

## Methods

### Cell culture

The human skin SCC cell line SRB12-p9 was derived by single cell cloning from SRB12 cells (a gift from Dr. Reuben Lotan, Department of Thoracic Head and Neck Medical Oncology, University of Texas M.D. Anderson Cancer Center). Cells were cultured in a humidified atmosphere at 5% CO_2_, in Dulbecco's Modified Eagle's Media-F12 supplemented with 10% fetal bovine serum.

### Severe combined immunodeficient (SCID) beige mice

SCID/bg mice were housed sterile cages in a temperature and humidity controlled AAALAC facility under a normal 12 hour light/dark cycle. All procedures were approved by LSUHSC Institutional Animal Care and Use Committee in accordance with NIH guidelines. The mice were allowed access to autoclaved food and water *ad libitum*.

### NF-κB-RE-luc (Oslo) mice

All mice were allowed food and water *ad libitum *and were housed according to institutional and NIH guidelines. Mice were purchased from the Xenogen Corporation (Alameda, California) and are bred and genotyped in our breeding colony. *NF-*κ*B-RE-luc *is a light producing animal model that carries a transgene containing 3 NF-κB response elements from the Igκ light chain promoter and modified firefly luciferase cDNA (Promega pGL-3) [23]. The reporter is expressed under basal conditions in the lymph node (neck), thymus (thoracic region), and Peyer's patches (abdominal region). Various inflammatory stimuli induce the reporter, such as LPS, TNF-*a*, and arthritis, which makes it suitable for the study of transcriptional regulation of NF-κB. Male and female mice (n = 3-4) were pre-treated with ACA (100 mg/kg bw), AUR (200 mg/kg bw) or vehicle (0.2 mL corn oil/25 g bw) once a day for 4 consecutive days. At 30 min after the last pretreatment, mice were injected with LPS (2.7 mg/kg bw). Mice were imaged on an IVIS 100 Imaging System at 30 min after the 3^rd ^pre-treatment dose as a pre-screen to ensure that the pre-treatments themselves did not activate NF-κB. Mice were imaged again at 3 h and 24 h after LPS treatment. Mice were injected with 2.5 mg D-luciferin substrate, along with ketamine/xylazine anesthetic, 15 minutes prior to each imaging. Luminescence intensity was quantified using Living Image (R) 3.0 software (Caliper Life Sciences, Inc., Hopkinton, MA).

### Xenograft Model

Groups of 5-8 female SCID/bg mice (6-12 weeks of age) each were fed control diets or diets containing ATRA (5, 10, 30 ppm), ACA (500 ppm) or AUR (1000 ppm) or ATRA + ACA/AUR, starting 1 week prior to tumor cell inoculation and for the duration of the study. Test diets were administered prior to the injection of tumor cells to be a chemoprevention protocol. In humans, this might be applicable to individuals who have been treated for a primary tumor, but are susceptible to the development of subsequent tumors. This could be a window of opportunity for chemoprevention. Mice were shaved (2 days prior to injection), then injected with 1 × 10^6 ^SRB12-p9 cells suspended in sterile PBS, s.c., palpated and weighed twice a week for 25-28 days following injection. Tumors were measured using digital calipers and tumor volume (mm^3^) was calculated using the following formula, based on the assumption of a near-spherical tumor shape: V = ((*l *+ *w*)/4)^3 ^* 1.33 * PI, where *l *= length, *w *= width. The studies were conducted at different times, so the timing was kept to a 25-28 day window for consistency. All mice in a particular study were euthanized on the same day so that comparisons could be made.

### microPET imaging

Tumor bearing mice were investigated by non-invasive imaging using a R4 microPET scanner (Siemens Medical Solutions USA, Inc., Knoxville, TN). In brief, the system operates in three dimensions and is composed of 6144 lutetium oxyorthosilicate crystal (LSO) detector elements, with a 7.8 cm axial and a 10 cm transaxial field-of-view. The [18F]-3'-fluoro-3'-deoxy-L-thymidine ([18F]FLT) radiotracer, a pyrimidine nucleoside analogue, has been validated as a promising PET radiopharmaceutical for monitoring tumor proliferation as well as response to therapy [24]. The [18F]FLT was synthesized using a 3-N-Boc-5'-O-dimethoxytrityl-3'-O-nosyl-thymidine precursor according to published method of Eisenhut *et al*. [25]. No-carrier-added [18F]FLT was administered intravenously by tail vein injection into experimental animals at a dose of 300 mCi/mouse. Sixty minutes after injection, the animals were anesthetized, positioned in the field of view of the scanner, and imaged for 15 minutes. Subsequently, the microPET images were reconstructed using an iterative reconstruction technique.

### Glutathione *S*-transferase (GST) and NAD(P)H quinone oxidoreductase (NQO) activities

Liver cytosolic GST activities were assayed spectrophotometrically (Shimadzu, Columbia, MD, kinetic mode) using two different substrates [26]. First, liver cytosol enriched fractions were prepared by differential centrifugation as previously described [27]. Protein concentrations were estimated using the Bradford method with BSA as a standard [28]. Aliquots of cytosolic samples were incubated with either 1 mM 1,2-dichloro-4-nitrobenzene (DCNB), 5 mM GSH in 0.1 M KH2PO4 buffer, pH 7.5; or with 1 mM 1-chloro-2,4-dinitrobenzene (CDNB), 1 mM GSH, in 0.1 M KH2PO4 buffer, pH 6.5. Changes in absorbances were assessed at 340 nm (CDNB) or 345 nm (DCNB), and enzyme activities were calculated using extinction coefficients of 9.6 nM^-1^/cm^-1 ^and 8.5 nM^-1^/cm^-1^, respectively [29]. Liver cytosolic NQO activity was assayed spectrophotometrically (kinetic mode) at 600 nm using 2,6-dichloroindophenol (1.25 mM) as a substrate and an extinction coefficient of 21 nM^-1^/cm^-1 ^as previously described [30].

### Statistical analysis

For NF-κB and GST data, differences between groups were compared using ANOVA followed by post-hoc analysis with Fisher's PLSD test and/or Tukey's test. Group comparisons are shown in the figure and table legends. Descriptive statistics of body weight and tumor volume were presented as means and least squared means along with standard deviation and sample size for a particular sub-group. Analysis of variance method was used to compare the body weight and tumor volume among groups (ACA, AUR and ATRA). Subsequently, the multiple comparison using Dunnett method was made among the group (all other group compares to control group). To perform the non-parametric data analysis, the body weight and tumor volume were ranked first and then similar analysis were performed as described above. A linear trend test was performed to assess the effect of ATRA dosage of 0, 5, 10, and 30 with/without ACA on the body weight and tumor volume at particular days (13, 15, 20, and 22) after treatment. Only these days have the same measurement for the dosage of ATRA, the number of mice in the control dosage level (0) were combination of two separated experiment in this study (n = 13), the number of mice are 8, 8, and 5 for dose level of 5, 10 and 30. All p-values, ≤ 0.05 were considered statistically significant. All statistical data analysis was performed by using SAS system 9.2 (SAS Inc, Gary, NC).

## Results

### ACA and AUR suppressed LPS-induced NF-κB activation in NF-κB-RE-luc (Oslo) mice

**Figure 1:**
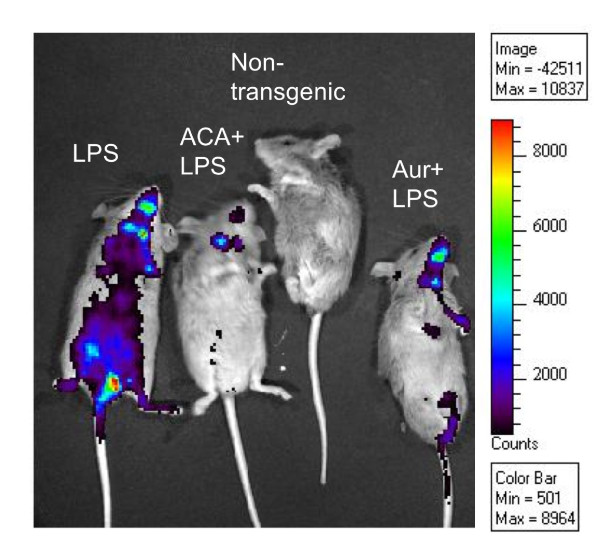
**ACA and AUR suppress LPS-induced NF-κB activation in NF-κB-RE-luc (Oslo) mice**. Picture depicts live animal imaging of luciferase.

**Figure 2:**
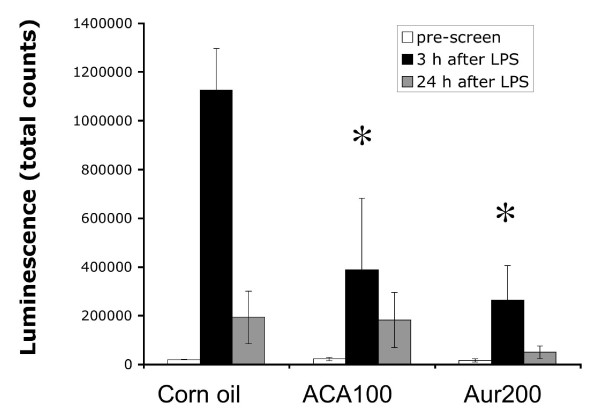
**Quantitation of luminescence activity in the NF-κB study**. Figures represent means ± SE (n = 3-4). * Significantly different from control at the same time-point (p ≤ 0.05) (ANOVA followed by Tukey's test).

Many phytochemicals suppress NF-κB activation, so we used a simple *in vivo *method using *NF-*κ*B-RE-luc (Oslo) *to first determine whether ACA and AUR also possessed this inhibitory activity. Male and female mice (n = 3-4) were pre-treated with ACA (100 mg/kg bw), AUR (200 mg/kg bw) or vehicle (0.2 mL corn oil/25 g bw) once a day for 4 consecutive days. On day 3, mice were imaged as a pre-screen to ensure that ACA and AUR did not activate the NF-κB reporter. On day 4, 30 min after the last pretreatment, mice were injected with LPS (2.7 mg/kg bw) and imaged 3 h and 24 h after LPS treatment, as described in materials and methods. Mice dosed with LPS expressed a significant increase in NF-κB luciferase at 3 h after LPS (Figures 1, 2). Both ACA and AUR significantly inhibited NF-κB luciferase expression by 65% and 76%, respectively at 3 h after LPS (Figure 2). There was very little luciferase expression at the pre-screen time point, confirming that the pre-treatments did not activate NF-κB. By 24 h, the expression of luciferase declined and there were no significant differences in any of the treatment groups.

### ACA and ATRA suppression of skin SCC tumor growth: A potentially beneficial combination

**Figure 3:**
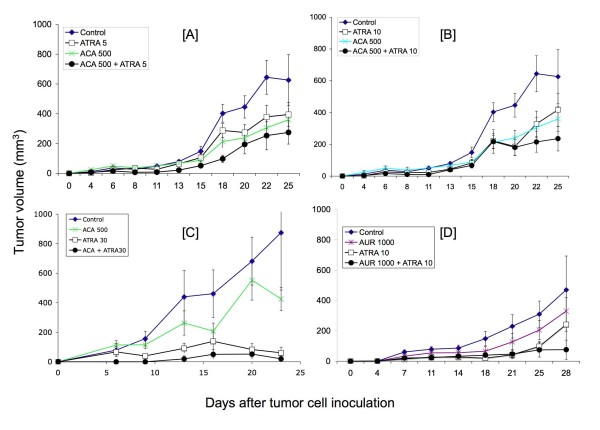
**Effects of combinations of ACA/AUR ± ATRA on SRB12-p9 SCC tumor growth in SCID/bg mice**. Groups of mice were fed diets containing the following combinations [A] ACA 500 ppm ± ATRA 5 ppm (n = 7-8); [B] ACA 500 ppm ± ATRA 10 ppm (n = 7-8); [C] ACA 500 ppm ± ATRA 30 ppm (n = 5); and [D] AUR 1000 ppm ± ATRA 10 ppm (n = 5). Figures represent means ± S.E. Note that the first two experiments (panels A & B) were conducted simultaneously, so the control and ACA groups are the same, but the two different doses of ATRA ± ACA were split into separate graphs for clarity.

**Figure 4:**
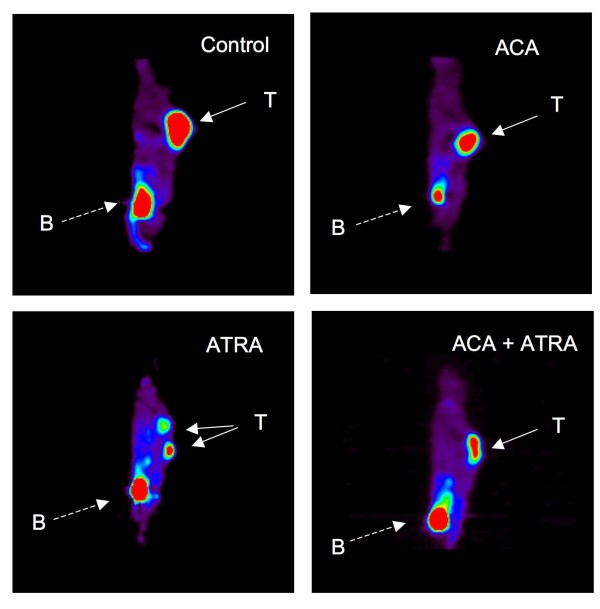
**Representative FLT-PET live animal imaging from the ACA 500 ± ATRA 30 study**. Solid arrows marked with "T" point to the tumors, and dashed arrows marked with "B" point to the urinary bladder, where the isotope is eliminated.

Previous data generated by our groups and others suggests that ATRA and ACA suppress Stat3 and NF-κB activation, respectively [7,22]. As both pathways have been shown to be important in the development of various cancers including skin SCC, we hypothesized that the simultaneous suppression of each pathway using ATRA + ACA would be more effective than using either agent alone at suppressing SCC growth. To address this hypothesis, female SCID/bg mice were inoculated with 1 × 10^6 ^SRB12-p9 cells and weighed and their tumors were measure by digital calipers over the course of 25 days. Groups of 5-8 mice each were fed control diets or diets containing ATRA (5, 10, and 30 ppm), ACA (500 ppm), or ATRA + ACA, starting 1 week prior to tumor cell inoculation and for the duration of the study. The dose of ACA was selected based on its activity in previous dietary studies [31-34]. Tumor volume increased rapidly at day 15 to day 25 after inoculation in the control diet groups, reaching a maximum of 650-875 mm^3^. ACA had a marginally significant effect on tumor volume between days 18-22 (Figures 3A, B) and [Additional file 1: Supplemental Tables S1, S2]. Dietary ATRA (5 ppm) modestly suppressed tumor volume but not significantly [Additional file 1: Supplemental Tables S1, S2]. Comparing both parametric and non-parametric analyses, the combination of 5 ppm ATRA and ACA in the diet significantly inhibited tumor volume starting at 11 days and continuing throughout the study, ranging from 56-76% inhibition [Additional file 1: Supplemental Tables S1, S2]. 10 ppm ATRA modestly inhibited tumor volume from 45-58% starting at day 11 up to day 22, but its effects were marginally significant - significant for a few days [Additional file 1: Supplemental Tables S1, S2]. The combination of 10 ppm ATRA and ACA significantly suppressed tumor volume by 50-67% starting at day 11 to day 25, again comparing both parametric and non-parametric analyses [Additional file 1: Supplemental Tables S1, S2]. In the 30 ppm ATRA experiment, ACA did not exhibit any significant effects on its own, although there was a 51% inhibition of tumor volume at day 23 that was not statistically significant. A lower number of subjects (5 mice instead of 7-8) in this experiment likely contributed to this lack of statistical significance, compared to the 5 and 10 ppm ATRA studies. However, 30 ppm ATRA strongly suppressed tumor volume by 70-93% from day 9 to day 23, and the combination of ATRA 30 with ACA significantly suppressed tumor volume from 89-98% (Figure 3C) and [Additional file 1: Supplemental Tables S1, S2]. Body weight analyses demonstrated a significant change in the ATRA 5 and 30 groups, for most time-points [Additional file 1: Supplemental Table S3]. However, dose-response analyses suggested that the effect was independent of ACA [Additional file 1: Supplemental Table S5]. Dose-response analyses of ATRA 5, 10, and 30 ppm ± ACA demonstrated relatively similar levels of significance, suggesting the effect on tumor volume was dependent on dose of ATRA [Additional file 1: Supplemental Table S4]. A linear trend analysis supported this observation [Additional file 1: Supplemental Table S6]. Live animal imaging (FLT) of representative mice from the 30 ppm ATRA combination study are shown in Figure 4. Sagittal sections through the tumor indicated that the size of tumors visualized in live mice corresponded with the values obtained from caliper measurements.

### AUR and ATRA suppression of skin SCC tumor growth: Another potentially beneficial combination

**Figure 5:**
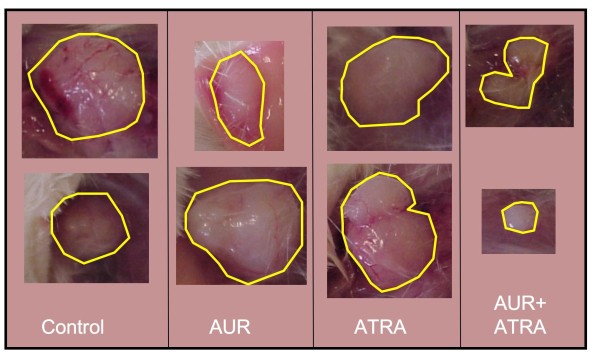
**Representative tumors from the Auraptene 1000 ± ATRA 10 study**. Photographs were taken from duplicate mice in each group. Lines were drawn around the tumor margins for clarity.

We next tested the AUR + ATRA combination in the skin SCC xenograft model. As shown in Figure 3D and [Additional file 1: Supplemental Table S7], treatment with AUR (1000 ppm in the diet) alone did not reduce tumor volume to a statistically significant level. 10 ppm ATRA, as observed in the first set of studies, significantly inhibited tumor volume by 49-87% starting at day 7 and continuing throughout the study (Figure 3D) and [Additional file 1: Supplemental Table S7]. The combination of AUR + 10 ppm ATRA also significantly inhibited tumor volume by 62-84% starting at day 7 and continuing until day 25. At day 28, the combination of ATRA + AUR suppressed tumor volume by 84%, although the results were not statistically significant at this date. Photos of tumors from representative mice illustrate this strong combination effect (Figure 5). Statistical analysis of body weights showed a modest reduction in body weights in the ATRA treated groups [Additional file 1: Supplemental Table S8].

Our laboratory and others have shown that one of the chemopreventive activities of AUR is induction of phase II detoxifying enzymes through activation of the Nrf-2/antioxidant response element [35,36]. In the current study, we also assessed whether the combination of AUR and ATRA maintained this effect. As shown in Table 1, AUR significantly induced GST activities using DCNB as a substrate. No significant effects on NQO activities or on GST activities using CDNB as a substrate were observed. CDNB is a more general substrate for GST activities [37], hence may have diluted out any specific effects of AUR at the dietary exposure level in the current study. Also, we have previously observed that AUR is also not a very strong inducer of hepatic NQO activity [35], so these results are not surprising. However, it appeared that ATRA itself might have modestly increased GST activity, and the combination of AUR and ATRA did not afford any increase in activity (using DCNB as a substrate) compared to ATRA alone, and no statistically significant increase compared to the vehicle control group. Thus ATRA may slightly inhibit the GST inducing effect of AUR.

**Table 1:** Effects of dietary combinations of AUR and ATRA on NQO1 and GST activities.

Diet	NQO1	CDNB * 1000	DCNB * 1000
Control	3.17 ± 0.27	316 ± 36	14.9 ± 2.0
AUR	4.09 ± 0.30	371 ± 47	27.5 ± 0.7**
ATRA	3.01 ± 0.50	371 ± 34	18.3 ± 1.5
AUR + ATRA	4.27 ± 0.57	386 ± 16	18.8 ± 1.6

** Significantly different from control diet (p < 0.01, Tukey's test)

Figures represent mean activities (μmol/min/mg protein) ± SE (n = 4-5).

## Discussion

Taken together, the current studies have demonstrated that ACA and AUR suppressed LPS-induced activation of NF-κB in *NF-κB-Re-luc *mice, and modestly inhibited SRB12-p9 tumor growth in the xenograft model. Although ATRA was fairly effective at doses as low as 10 ppm in the diet, the inhibitory effect can be maintained to the end of the study when combined with either ACA or AUR. Although neither ACA nor AUR possessed strong inhibitory effects on their own, these data suggest that they may be useful in combination with ATRA. This is particularly important because ATRA is known to produce hypervitaminosis A-related side effects, and if its dose can be limited and efficacy improved by combining with naturally occurring dietary agents, these combinations could be clinically useful. However, diets containing ATRA led to a dose-related decrease in body weights, which was not different when combined with ACA or AUR.

Many other phytochemicals are known to suppress NF-κB activation [38]. Among these, several are related to the Zingiberaceae family, including gingerol and curcumin. These agents have been known to suppress many types of cancer, including that of skin. Taken together, these data lend to an intriguing suggestion that suppression of NF-κB activation in combination with suppression of the B-raf/Mek/Erk/Stat3 pathway may be a useful strategy in the suppression of cancer progression. Many downstream signaling effectors involved in malignancy are regulated by both Stat3 and NF-κB, such that targeting both of these signaling pathways at once by combining ATRA + AUR, may produce a synergistic tumor suppressive effect. New evidence is also helping to decipher the connections between NF-κB and STAT3. In 2007, Yang and associates discovered that unphosphorylated STAT3 competes with IKB for binding to unphosphorylated NF-κB. This is turn results in nuclear localization of this complex, leading to activation of NF-κB regulated genes [39]. Most recently, Lee and co-workers demonstrated that STAT3 impairs nuclear export of NF-κB *via *activation of RelA acetylation [40]. Thus it appears that STAT3 may enhance the effects of NF-κB through multiple mechanisms.

The combinatorial effects of ATRA and AUR were encouraging. AUR is known to activate Nrf2/ARE and induces carcinogen and oxidant-detoxifying enzymes. However, recently, a "dark side" of Nrf2 has been suggested: that Nrf2 is upregulated in cancer (particularly lung cancer) and provides cancer cells with protection against the hostile tumor microenvironment [41]. In fact, shRNA against Nrf2 in lung cancer cells attenuated their growth in mouse xenografts compared to vector control treated cells [42]. It is argued that chemopreventive agents that increase Nrf2 activity may produce chemoresistance to therapy [43]. However, Wang and colleagues demonstrated that ATRA reduced binding of Nrf2 to the ARE enhancer [42]. ATRA has been reputed to possess pro-oxidant activities at therapeutic concentrations. If it suppresses Nrf2 activation it may render cells more susceptible to oxidative stress. In fact, Tan and colleagues confirmed that ATRA activates the Nrf2/ARE transcription pathway [44]. Although beyond the scope of the present study, it appears that perhaps ATRA behaves in combination with AUR as a partial agonist: that is, ATRA has a mild effect on activating Nrf2 by itself, but is milder than AUR, and in combination, may mitigate the effects of AUR, leading to an intermediate effect on Nrf2/ARE. Considering the potentially controversial role of Nrf2/ARE in carcinogenesis, we believe that the combination of ATRA with AUR for cancer therapy is even more enticing, because both agents were shown to be more effective at suppressing tumor growth in combination, despite the apparently intermediate effect on Nrf2/ARE.

## Conclusions

In conclusion, we report that the combination of two different phytochemicals, ACA and AUR in the diet, enhanced the chemoprotective effect of ATRA against human skin cancer cell growth in a xenograft model. In particular, AUR appeared to be synergistic with the effects of ATRA, whereas ACA appeared to prolong the tumor suppressive effects of ATRA. We believe these mechanisms to be related to the suppression of at least two pathways, Stat3 and NF-κB. Future studies may be warranted to capitalize on using these combinations to target multiple oncogenic pathways to enhance efficacy and lower potential off-target effects.

## Abbreviations

ACA: 1'-acetoxychavicol acetate; ARE: antioxidant response element; ATRA: all-trans retinoic acid; AUR: auraptene; CDNB: 1-chloro-2,4-dinitrobenzene; DCNB: 1,2-dichloro-4-nitrobenzene; GST: glutathione S-transferase; LPS: lipopolysaccharide; NF-κB: nuclear factor kappa-B; NMSC: nonmelanoma skin cancer; NQO: NAD(P)H quinone oxidoreductase; SCC: Squamous cell carcinoma; SCID: severe combined immunodeficiency; TPA: 12-*O*-tetradecanoylphorbol-13-acetate.

## Competing interests

The authors declare that they have no competing interests.

## Authors' contributions

The research team was directed equally by HK and JC. HK and MJM carried out the NF-κB-RE-luc mouse study. JC, ZS, DR, AR, JR, and HK conducted the tumor studies in the SCID mice. HK and MP conducted the CDNB, DCNB, and NQO assays. RS conducted the statistical analyses. PA analyzed the tissues for histopathology. The manuscript was written by HK with the assistance of AR, DR, MJM, RS, and JC. All the authors have seen the manuscript and agree to its contents.

## Authors' information

HK and JC are Associate professors (Ph.D.); RS is an Associate professor of Medicine (Ph.D.); MJM is a Professor (Ph.D.); PA is a Professor of Pathology (M.D.). ZS and DR were Ph.D. students during the study, and AR was a summer undergraduate intern. MP and JR are Research Associates/Lab Managers (B.S.).

## Pre-publication history

The pre-publication history for this paper can be accessed here:

http://www.biomedcentral.com/1471-2407/10/394/prepub

## Supplementary Material

Additional file 1 **Supplemental Table S1**. 2 × 2 Factorial design for statistical analyses. Supplemental Table S2. Tumor volume (TV) statistics for ACA ± ATRA xenograft study. Supplemental Table S3. Body weight (BW) statistics for ACA ± ATRA xenograft study. Supplemental Table S4: Tumor volume (TV) as a function of ATRA dose: statistics for ACA ± ATRA xenograft study. Supplemental Table S5: Body weight (BW) statistics for ACA ± ATRA xenograft study as a function of ATRA dose. Supplemental Table S6: Trend effect of ATRA dose in presence or absence of ACA on tumor volume (TV). Supplemental Table S7: Tumor volume (TV) statistics for AUR ± ATRA study. Supplemental Table S8: Body weight (BW) data for AUR ± ATRA study. Click here for file
