# Supplementary material for: Effects of ATRA combined with citrus and ginger-derived compounds in human SCC xenografts
Source: BMC Cancer. 2010 Jul 26;10:394. doi: 10.1186/1471-2407-10-394 (PMC2916922; doi:10.1186/1471-2407-10-394)
Supplement: Additional file 1 — Supplemental Table S1. 2 × 2 Factorial design for statistical analyses. Supplemental Table S2. Tumor volume (TV) statistics for ACA ± ATRA xenograft study. Supplemental Table S3. Body weight (BW) statistics for ACA ± ATRA xenograft study. Supplemental Table S4: Tumor volume (TV) as a function of ATRA dose: statistics for ACA ± ATRA xenograft study. Supplemental Table S5: Body weight (BW) statistics for ACA ± ATRA xenograft study as a function of ATRA dose. Supplemental Table S6: Trend effect of ATRA dose in presence or absence of ACA on tumor volume (TV). Supplemental Table S7: Tumor volume (TV) statistics for AUR ± ATRA study. Supplemental Table S8: Body weight (BW) data for AUR ± ATRA study. [file 1471-2407-10-394-S1.PDF]

Supplemental Table 1

2x2 Factorial design for statistical analyses

		ACA or AUR	
		No	Yes
ATRA	No	Group 1	Group 2
	Yes	Group 3	Group 4

Supplemental Table 2

Tumor volume (TV) statistics for ACA ± ATRA xenograft study*

dose of ATRA in study	group	days	TV (mm3) mean	TV (mm3) median	SD	N	** paramet ProbtDiff	*** non-par ProbtDiff
5	1	d4	12.25	0.00	20.75	8		
5	1	d6	37.58	38.27	21.48	8		
5	1	d8	27.27	25.75	17.49	8		
5	1	d11	49.24	51.90	21.93	8		
5	1	d13	79.76	71.26	32.04	8		
5	1	d15	148.94	158.02	93.17	8		
5	1	d18	403.24	417.46	166.76	8		
5	1	d20	445.99	538.27	211.11	8		
5	1	d22	644.77	656.43	320.53	8		
5	1	d25	625.79	523.27	484.29	8		
5	2	d4	24.90	20.53	28.04	7	0.41441	0.58905
5	2	d6	50.28	40.95	36.00	7	0.62947	0.80312
5	2	d8	38.19	28.66	48.36	7	0.879387	0.97526
5	2	d11	50.65	49.52	34.41	7	0.998387	0.99571
5	2	d13	67.92	88.57	38.53	7	0.766575	0.86162
5	2	d15	88.28	95.46	45.09	7	0.209158	0.53903
5	2	d18	213.69	262.43	109.45	7	0.020304	0.10473
5	2	d20	239.54	252.65	130.68	7	0.070846	0.17356
5	2	d22	306.13	343.93	170.82	7	0.041873	0.07864
5	2	d25	360.29	413.37	209.65	7	0.255251	0.63761
5	3	d4	4.19	0.00	7.51	8	0.712055	0.9413
5	3	d6	24.45	28.19	22.34	8	0.582512	0.58359
5	3	d8	36.16	17.35	44.10	8	0.922112	0.95213
5	3	d11	27.76	27.89	8.84	8	0.121273	0.09392
5	3	d13	65.05	70.00	24.67	8	0.614183	0.62824
5	3	d15	106.07	94.02	69.84	8	0.441469	0.69989
5	3	d18	288.45	259.97	137.53	8	0.193956	0.33445
5	3	d20	275.04	315.42	146.74	8	0.137991	0.28154
5	3	d22	379.38	394.27	221.93	8	0.115741	0.23454
5	3	d25	395.53	429.89	227.07	8	0.334857	0.78862
5	4	d4	7.89	0.00	11.18	8	0.934079	0.99323
5	4	d6	16.56	18.90	15.11	8	0.226784	0.16254
5	4	d8	7.68	0.00	21.72	8	0.552867	0.11544
5	4	d11	9.00	6.31	9.92	8	0.001792	0.00014
5	4	d13	22.52	28.61	15.92	8	0.001258	0.00074
5	4	d15	52.35	52.43	40.65	8	0.019131	0.03109
5	4	d18	97.97	80.17	70.87	8	0.000145	0.00017
5	4	d20	195.02	138.40	180.39	8	0.018457	0.03481
5	4	d22	253.86	179.25	269.69	8	0.013103	0.01764
5	4	d25	275.20	285.36	219.62	8	0.081916	0.16803
10	1	d4	12.25	0.00	20.75	8		
10	1	d6	37.58	38.27	21.48	8		
10	1	d8	27.27	25.75	17.49	8		

10	1	d11	49.24	51.90	21.93	8		
10	1	d13	79.76	71.26	32.04	8		
10	1	d15	148.94	158.02	93.17	8		
10	1	d18	403.24	417.46	166.76	8		
10	1	d20	445.99	538.27	211.11	8		
10	1	d22	644.77	656.43	320.53	8		
10	1	d25	625.79	523.27	484.29	8		
10	2	d4	24.90	20.53	28.04	7	0.379599	0.60353
10	2	d6	50.28	40.95	36.00	7	0.654623	0.84357
10	2	d8	38.19	28.66	48.36	7	0.846836	0.96661
10	2	d11	50.65	49.52	34.41	7	0.998567	0.99553
10	2	d13	67.92	88.57	38.53	7	0.763394	0.83023
10	2	d15	88.28	95.46	45.09	7	0.179477	0.51531
10	2	d18	213.69	262.43	109.45	7	0.076889	0.0906
10	2	d20	239.54	252.65	130.68	7	0.059012	0.23831
10	2	d22	306.13	343.93	170.82	7	0.025918	0.10868
10	2	d25	360.29	413.37	209.65	7	0.287702	0.54281
10	3	d4	2.30	0.00	4.26	8	0.539325	0.69667
10	3	d6	26.43	21.74	26.41	8	0.714529	0.64804
10	3	d8	21.57	0.00	31.39	8	0.96962	0.51162
10	3	d11	22.96	23.47	11.46	8	0.057607	0.02555
10	3	d13	44.08	50.12	24.05	8	0.048101	0.06595
10	3	d15	82.38	73.50	54.26	8	0.111426	0.25709
10	3	d18	221.95	244.31	130.13	8	0.080623	0.12595
10	3	d20	186.64	148.48	158.07	8	0.01116	0.02484
10	3	d22	328.75	243.15	226.98	8	0.032189	0.11127
10	3	d25	417.38	404.91	293.96	8	0.447798	0.7468
10	4	d4	3.74	0.00	7.34	8	0.650271	0.80262
10	4	d6	16.15	18.96	14.46	8	0.239413	0.15701
10	4	d8	10.42	0.00	24.29	8	0.59146	0.14753
10	4	d11	9.75	0.00	13.57	8	0.003156	0.00074
10	4	d13	40.43	37.00	15.64	8	0.027039	0.02114
10	4	d15	66.94	57.15	45.33	8	0.039851	0.106
10	4	d18	217.64	140.25	209.24	8	0.072083	0.07862
10	4	d20	180.66	129.10	145.38	8	0.009352	0.02962
10	4	d22	215.26	126.29	184.01	8	0.003076	0.00879
10	4	d25	236.24	175.38	220.15	8	0.06103	0.11973
30	1	d6	79.02	47.59	100.37	5		
30	1	d8	179.39	157.09	111.07	5		
30	1	d13	440.16	298.64	400.99	5		
30	1	d15	461.34	530.16	360.73	5		
30	1	d20	681.95	743.65	363.37	5		
30	1	d22	875.81	499.14	872.30	5		
30	2	d6	113.52	127.51	66.07	5	0.783037	0.572
30	2	d8	115.09	150.16	58.63	5	0.295169	0.65875
30	2	d13	262.91	190.91	184.12	5	0.477229	0.98491
30	2	d15	207.85	186.93	121.80	5	0.164716	0.71331
30	2	d20	553.57	546.15	303.14	5	0.74447	0.9172
30	2	d22	426.44	413.37	175.58	5	0.293949	0.93909

30	3	d6	67.26	47.59	74.63	5	0.987144	0.99974
30	3	d8	39.62	36.00	25.33	5	0.008847	0.01875
30	3	d13	91.17	101.91	77.97	5	0.06566	0.05954
30	3	d15	138.86	124.48	140.01	5	0.062675	0.23574
30	3	d20	82.98	63.35	89.21	5	0.003585	0.00026
30	3	d22	61.06	32.19	85.12	5	0.028189	0.00214
30	4	d6	0.00	0.00	0.00	5	0.222349	0.03894
30	4	d8	0.00	0.00	0.00	5	0.00117	3.8E-05
30	4	d13	19.86	0.00	44.40	5	0.024502	0.00132
30	4	d15	50.58	0.00	71.87	5	0.016284	0.02001
30	4	d20	51.92	0.00	76.02	5	0.002362	9.6E-05
30	4	d22	20.11	24.37	13.31	5	0.021045	0.00027

*Group identification

	ACA	ATRA
1	no	no
2	yes	no
3	no	yes
4	yes	yes

**parametric statistics

***non-parametric statistics

*All p-values are comparisons to the control group (1)

light blue: marginally significant p-value

dark blue: significant p-value

Supplemental Table 3

Body weight (BW) statistics for ACA ± ATRA xenograft study*

dose of ATRA in study	group	days	BW (g) mean	BW (g) median	SD	N	** para ProbtDiff	*** non-para ProbtDiff
5	1	d-6	21.5	22.0	1.4	8		
5	1	d-3	20.8	20.5	1.5	8		
5	1	d-1	20.5	20.5	1.3	8		
5	1	d0	21.3	21.0	1.5	8		
5	1	d2	20.9	20.5	1.0	8		
5	1	d4	21.4	21.5	1.4	8		
5	1	d6	21.6	22.0	1.5	8		
5	1	d8	22.1	22.0	1.6	8		
5	1	d11	21.3	21.0	1.5	8		
5	1	d13	22.0	22.1	1.3	8		
5	1	d15	23.0	22.5	1.2	8		
5	1	d18	21.6	22.0	1.3	8		
5	1	d20	22.8	22.5	1.5	8		
5	1	d22	23.5	24.0	1.4	8		
5	1	d25	23.2	23.4	1.8	8		
5	2	d-6	20.5	20.0	2.3	8	0.590321	0.33983
5	2	d-3	21.4	22.0	2.7	8	0.828769	0.96413
5	2	d-1	19.6	20.0	1.5	8	0.456322	0.35068
5	2	d0	21.0	21.0	2.3	7	0.98868	0.94924
5	2	d2	20.3	20.0	2.4	7	0.834546	0.32377
5	2	d4	21.0	21.0	2.6	7	0.959789	0.70416
5	2	d6	20.0	20.0	2.2	7	0.211453	0.12754
5	2	d8	21.7	22.0	2.1	7	0.951432	0.66074
5	2	d11	20.7	20.0	1.8	7	0.83612	0.67183
5	2	d13	21.6	21.7	1.9	7	0.92598	0.72181
5	2	d15	21.6	22.0	2.3	7	0.217305	0.07558
5	2	d18	20.9	20.0	2.0	7	0.701734	0.53646
5	2	d20	21.1	22.0	2.0	7	0.163364	0.15359
5	2	d22	22.0	22.0	1.8	7	0.193156	0.11423
5	2	d25	22.5	23.1	2.0	7	0.81089	0.62274
5	3	d-6	20.6	20.0	1.2	8	0.680263	0.54684
5	3	d-3	19.5	20.0	1.1	8	0.383555	0.32172
5	3	d-1	17.9	18.0	1.2	8	0.002052	0.0012
5	3	d0	20.0	20.0	1.5	8	0.428132	0.38569
5	3	d2	19.4	19.5	1.1	8	0.194214	0.05003
5	3	d4	20.6	20.0	0.9	8	0.751673	0.6622
5	3	d6	20.6	20.0	1.2	8	0.55074	0.44701
5	3	d8	20.5	20.0	1.1	8	0.218278	0.05387
5	3	d11	19.1	19.5	1.0	8	0.024431	0.00916
5	3	d13	20.0	19.9	1.1	8	0.036995	0.0065
5	3	d15	19.4	20.0	0.9	8	0.000267	2.5E-05
5	3	d18	20.0	20.0	1.5	8	0.145158	0.08991
5	3	d20	20.8	20.5	1.3	8	0.054136	0.03621

5	3	d22	21.3	21.5	0.9	8	0.024101	0.00852
5	3	d25	21.4	21.6	1.4	8	0.151837	0.11557
5	4	d-6	19.0	20.0	2.4	8	0.034671	0.03671
5	4	d-3	19.8	20.0	1.6	8	0.555903	0.58415
5	4	d-1	19.1	20.0	1.5	8	0.13852	0.13757
5	4	d0	19.5	20.0	2.2	8	0.183407	0.27653
5	4	d2	18.0	18.0	1.9	8	0.004925	0.00094
5	4	d4	19.0	19.5	2.1	8	0.038835	0.02619
5	4	d6	18.5	19.0	2.1	8	0.004474	0.00339
5	4	d8	19.3	20.0	2.4	8	0.012719	0.00573
5	4	d11	18.5	18.5	1.7	8	0.00323	0.00197
5	4	d13	19.5	19.8	1.8	8	0.008726	0.00407
5	4	d15	19.6	20.0	1.7	8	0.000617	0.0002
5	4	d18	18.9	19.0	1.8	8	0.006956	0.00419
5	4	d20	19.4	20.0	1.8	8	0.000868	0.0007
5	4	d22	19.3	20.0	2.1	8	3.79E-05	1.2E-05
5	4	d25	20.4	20.9	2.1	8	0.012421	0.01736
10	1	d-6	21.5	22.0	1.4	8		
10	1	d-3	20.8	20.5	1.5	8		
10	1	d-1	20.5	20.5	1.3	8		
10	1	d0	21.3	21.0	1.5	8		
10	1	d2	20.9	20.5	1.0	8		
10	1	d4	21.4	21.5	1.4	8		
10	1	d6	21.6	22.0	1.5	8		
10	1	d8	22.1	22.0	1.6	8		
10	1	d11	21.3	21.0	1.5	8		
10	1	d13	22.0	22.1	1.3	8		
10	1	d15	23.0	22.5	1.2	8		
10	1	d18	21.6	22.0	1.3	8		
10	1	d20	22.8	22.5	1.5	8		
10	1	d22	23.5	24.0	1.4	8		
10	1	d25	23.2	23.4	1.8	8		
10	2	d-6	20.5	20.0	2.3	8	0.563939	0.43688
10	2	d-3	21.4	22.0	2.7	8	0.877651	0.88979
10	2	d-1	19.6	20.0	1.5	8	0.484318	0.40511
10	2	d0	21.0	21.0	2.3	7	0.985809	0.96917
10	2	d2	20.3	20.0	2.4	7	0.859761	0.53961
10	2	d4	21.0	21.0	2.6	7	0.962208	0.82167
10	2	d6	20.0	20.0	2.2	7	0.20046	0.16557
10	2	d8	21.7	22.0	2.1	7	0.934417	0.73186
10	2	d11	20.7	20.0	1.8	7	0.874947	0.76869
10	2	d13	21.6	21.7	1.9	7	0.937013	0.72489
10	2	d15	21.6	22.0	2.3	7	0.410326	0.18936
10	2	d18	20.9	20.0	2.0	7	0.778343	0.64508
10	2	d20	21.1	22.0	2.0	7	0.208947	0.22688
10	2	d22	22.0	22.0	1.8	7	0.260568	0.16709
10	2	d25	22.5	23.1	2.0	7	0.808127	0.75687
10	3	d-6	21.0	20.5	1.9	8	0.903555	0.86893
10	3	d-3	21.1	21.0	2.0	8	0.968512	0.94377

10	3	d-1	19.4	20.0	0.9	8	0.290322	0.16451
10	3	d0	21.4	22.0	1.2	8	0.997943	0.97524
10	3	d2	19.9	20.0	1.5	8	0.548245	0.47398
10	3	d4	21.0	21.5	1.6	8	0.958434	0.99405
10	3	d6	20.3	20.0	1.3	8	0.292116	0.25034
10	3	d8	20.6	21.0	1.3	8	0.197909	0.176
10	3	d11	19.1	19.5	1.8	8	0.047757	0.05279
10	3	d13	20.7	20.7	1.5	8	0.261311	0.28078
10	3	d15	21.4	22.0	1.9	8	0.285187	0.22202
10	3	d18	20.1	20.0	2.0	8	0.287815	0.25034
10	3	d20	21.0	21.0	1.8	8	0.138412	0.11741
10	3	d22	21.3	21.5	1.8	8	0.044334	0.02481
10	3	d25	22.2	22.1	1.5	8	0.591742	0.61847
10	4	d-6	20.1	20.0	1.6	8	0.318621	0.32199
10	4	d-3	21.6	22.0	2.0	8	0.734853	0.68424
10	4	d-1	19.6	20.0	1.8	8	0.484318	0.54923
10	4	d0	21.0	21.5	1.9	8	0.984329	0.99229
10	4	d2	20.0	20.5	2.1	8	0.643162	0.79566
10	4	d4	20.5	21.0	1.8	8	0.674854	0.80694
10	4	d6	19.3	20.0	1.9	8	0.030588	0.02757
10	4	d8	21.1	22.0	1.6	8	0.500171	0.64994
10	4	d11	19.8	20.0	1.7	8	0.207359	0.18856
10	4	d13	19.7	20.1	1.9	8	0.028327	0.0346
10	4	d15	20.5	21.0	2.6	8	0.056078	0.05477
10	4	d18	19.6	20.0	2.3	8	0.111459	0.06306
10	4	d20	20.1	20.5	1.8	8	0.015963	0.01861
10	4	d22	19.1	19.5	2.0	8	0.000101	7.8E-05
10	4	d25	21.1	21.0	1.9	8	0.068653	0.09232
30	1	d-3	20.8	20.0	1.1	5		
30	1	d-1	21.2	21.0	0.8	5		
30	1	d2	20.4	20.0	0.9	5		
30	1	d4	20.8	20.0	1.1	5		
30	1	d6	21.2	21.0	0.8	5		
30	1	d8	22.6	22.0	0.9	5		
30	1	d11	22.2	22.0	1.1	5		
30	1	d13	21.0	21.0	1.0	5		
30	1	d15	22.4	22.0	0.5	5		
30	1	d20	21.2	20.0	1.8	5		
30	1	d22	23.0	22.8	0.9	5		
30	2	d-3	18.4	18.0	2.2	5	0.17349	0.21288
30	2	d-1	19.4	20.0	1.9	5	0.191628	0.24828
30	2	d2	18.6	19.0	2.6	5	0.259004	0.24082
30	2	d4	20.0	20.0	2.4	5	0.807829	0.501
30	2	d6	19.6	20.0	1.7	5	0.158115	0.16061
30	2	d8	19.4	20.0	1.9	5	0.033491	0.01387
30	2	d11	21.4	22.0	1.9	5	0.767727	0.62621
30	2	d13	19.4	19.0	1.7	5	0.226107	0.15115
30	2	d15	20.2	20.0	1.8	5	0.119795	0.06521
30	2	d20	19.2	19.0	1.9	5	0.236751	0.12241

30	2	d22	21.7	21.9	1.9	5	0.610401	0.21447
30	3	d-3	20.8	21.0	1.9	5	1	0.99998
30	3	d-1	20.2	20.0	1.3	5	0.613797	0.5539
30	3	d2	19.8	20.0	1.3	5	0.899511	0.82575
30	3	d4	20.0	20.0	1.4	5	0.807829	0.71968
30	3	d6	20.4	21.0	1.5	5	0.644754	0.64776
30	3	d8	20.2	21.0	2.2	5	0.126391	0.05882
30	3	d11	20.4	21.0	1.5	5	0.213612	0.12621
30	3	d13	18.8	20.0	1.8	5	0.070332	0.05698
30	3	d15	20.8	22.0	1.8	5	0.313596	0.17909
30	3	d20	19.2	20.0	1.3	5	0.236751	0.17243
30	3	d22	20.7	21.9	2.8	5	0.18868	0.11414
30	4	d-3	21.0	22.0	2.4	5	0.997012	0.99777
30	4	d-1	20.2	20.0	1.8	5	0.613797	0.62579
30	4	d2	17.6	18.0	1.5	5	0.049284	0.02584
30	4	d4	17.6	18.0	1.7	5	0.026059	0.0164
30	4	d6	16.6	16.0	0.9	5	9.99E-05	0.00045
30	4	d8	16.6	16.0	1.9	5	0.000227	0.00014
30	4	d11	18.4	18.0	1.7	5	0.00445	0.00296
30	4	d13	16.4	16.0	1.1	5	0.000332	0.00031
30	4	d15	18.0	18.0	2.0	5	0.001673	0.00159
30	4	d20	15.6	14.0	2.2	5	0.000508	0.00062
30	4	d22	18.0	17.4	1.8	5	0.002631	0.00129

*Group identification

	ACA	ATRA
1	no	no
2	yes	no
3	no	yes
4	yes	yes

**parametric statistics
***non-parametric statistics

*All p-values are comparisons to the control group (1)
light blue: marginally significant p-value
dark blue: significant p-value

Supplemental Table 4

Tumor volume (TV) as a function of ATRA dose: statistics for ACA ± ATRA xenograft study*

ACA	ATRA dose	N	Days on study	Mean TV (mm3)	Median TV (mm3)	SD	LSMean	para** ProbtDiff	nonpara*** ProbtDiff
No	0	13	d13	218.38	111.13	295.80	48.54		
No	5	8	d13	65.05	70.00	24.67	37.38	0.46747	0.303788
No	10	8	d13	44.08	50.12	24.05	26.13	0.01093	0.009986
No	30	5	d13	91.17	101.91	77.97	38.90	0.77328	0.548628
Yes	0	12	d13	149.16	102.03	152.38	47.29	0.99999	
Yes	5	8	d13	22.52	28.61	15.92	14.50	3.8E-05	0.442545
Yes	10	8	d13	40.43	37.00	15.64	23.88	0.00405	0.139606
Yes	30	5	d13	19.86	0.00	44.40	14.00	0.0004	0.639179
No	0	13	d15	269.10	171.95	271.04	47.31		
No	5	8	d15	106.07	94.02	69.84	35.50	0.59517	0.095155
No	10	8	d15	82.38	73.50	54.26	29.44	0.16794	0.00183
No	30	5	d15	138.86	124.48	140.01	36.60	0.82201	0.000145
Yes	0	12	d15	138.10	116.91	101.46	40.42	0.91076	
Yes	5	8	d15	52.35	52.43	40.65	20.44	0.00996	0.380269
Yes	10	8	d15	66.94	57.15	45.33	26.13	0.06631	0.132737
Yes	30	5	d15	50.58	0.00	71.87	20.60	0.04	0.000176
No	0	13	d20	536.74	554.26	290.32	51.96		
No	5	8	d20	275.04	315.42	146.74	37.56	0.23715	8.96E-05
No	10	8	d20	186.64	148.48	158.07	27.00	0.00532	0.004128
No	30	5	d20	82.98	63.35	89.21	15.70	0.00034	0.000522
Yes	0	12	d20	370.38	313.00	262.45	42.38	0.54958	
Yes	5	8	d20	195.02	138.40	180.39	27.88	0.00773	0.02917
Yes	10	8	d20	180.66	129.10	145.38	27.50	0.0066	0.154792
Yes	30	5	d20	51.92	0.00	76.02	11.20	4.9E-05	0.073086
No	0	12	d22	733.63	579.07	572.06	50.65		
No	5	8	d22	379.38	394.27	221.93	40.69	0.62776	0.178366
No	10	8	d22	328.75	243.15	226.98	36.50	0.24989	0.162818
No	30	5	d22	61.06	32.19	85.12	12.70	0.00016	0.004733
Yes	0	12	d22	356.26	403.48	175.97	37.88	0.23734	
Yes	5	8	d22	253.86	179.25	269.69	28.63	0.01738	0.474923
Yes	10	8	d22	215.26	126.29	184.01	27.13	0.00942	0.353239
Yes	30	5	d22	20.11	24.37	13.31	7.60	1.7E-05	0.003603

*All p-values are comparisons to the control group (No ACA, No ATRA)
light blue: marginally significant p-value
dark blue: significant p-value
**parametric statistics
***non-parametric statistics

Supplemental Table 5

Body weight (BW) as a function of ATRA dose: statistics for ACA ± ATRA xenograft study*

ACA	ATRA dose	N	Days on study	Mean (g)	Median (g)	SD	LSMean	para** ProbtDiff	nonpara*** ProbtDiff
No	0	13	d13	21.62	21.80	1.24	21.62		
No	5	8	d13	19.96	19.90	1.15	19.96	0.03155	0.014906
No	10	8	d13	20.65	20.70	1.47	20.65	0.30496	0.379505
No	30	5	d13	18.80	20.00	1.79	18.80	0.00136	0.001611
Yes	0	12	d13	20.68	20.40	2.06	22.77		
Yes	5	8	d13	19.49	19.80	1.81	19.38	1.8E-05	6.08E-06
Yes	10	8	d13	19.74	20.10	1.91	21.38	0.08559	0.075775
Yes	30	5	d13	16.40	16.00	1.14	20.80	0.0303	0.025649
No	0	13	d15	22.77	22.00	1.01	22.15		
No	5	8	d15	19.38	20.00	0.92	20.75	0.15388	0.181835
No	10	8	d15	21.38	22.00	1.92	21.00	0.28764	0.373295
No	30	5	d15	20.80	22.00	1.79	19.20	0.00399	0.002748
Yes	0	12	d15	21.00	20.00	2.13	23.32		
Yes	5	8	d15	19.63	20.00	1.69	21.25	0.02091	0.005049
Yes	10	8	d15	20.50	21.00	2.56	21.25	0.02091	0.006995
Yes	30	5	d15	18.00	18.00	2.00	20.68	0.01108	0.01999
No	0	13	d20	22.15	22.00	1.72	20.68		
No	5	8	d20	20.75	20.50	1.28	19.49	0.39403	0.436393
No	10	8	d20	21.00	21.00	1.77	19.74	0.58011	0.721018
No	30	5	d20	19.20	20.00	1.30	16.40	0.00048	0.0048
Yes	0	12	d20	20.33	20.00	2.10	21.00		
Yes	5	8	d20	19.38	20.00	1.77	19.63	0.39145	0.406392
Yes	10	8	d20	20.13	20.50	1.81	20.50	0.92901	0.996183
Yes	30	5	d20	15.60	14.00	2.19	18.00	0.03638	0.064518
No	0	13	d22	23.32	23.40	1.23	20.33		
No	5	8	d22	21.25	21.50	0.89	19.38	0.61263	0.550974
No	10	8	d22	21.25	21.50	1.75	20.13	0.99253	0.999535
No	30	5	d22	20.68	21.90	2.81	15.60	0.00029	0.011992
Yes	0	12	d22	21.88	21.95	1.78	21.88		
Yes	5	8	d22	19.25	20.00	2.05	19.25	0.01518	0.006378
Yes	10	8	d22	19.13	19.50	2.03	19.13	0.01065	0.005792
Yes	30	5	d22	17.96	17.40	1.82	17.96	0.00177	0.00116

*All p-values are comparisons to the control group (No ACA, No ATRA)
light blue: marginally significant p-value
dark blue: significant p-value
**parametric statistics
***non-parametric statistics

Supplemental Table 6

Trend effect of ATRA dose in presence or absence of ACA on tumor volume (TV)*

ACA	Days on study	DF	SS	MS	FValue	ProbF
No	d13	1	59103	59103	1.637755	0.210442
No	d15	1	62620	62620	1.851335	0.183764
No	d20	1	8E+05	8E+05	16.79518	0.000291
No	d22	1	2E+06	2E+06	10.03988	0.003511
Yes	d13	1	48893	48893	5.314928	0.028492
Yes	d15	1	21961	21961	3.984085	0.055399
Yes	d20	1	3E+05	3E+05	8.421949	0.007011
Yes	d22	1	4E+05	4E+05	10.44137	0.003063

*Tumor volume (TV, mm3)
*light blue: marginally significant p-value
*dark blue: significant p-value

DF: degree of freedom
SS: sum of square
MS: Mean Square
Fvalue: Value of F test
PorbF: P-value

Supplemental Table 7

Tumor volume (TV) statistics for AUR ± ATRA xenograft study*

Treatment group	group	days on study	TV (mm3) mean	TV (mm3) median	SD	N	** paramet ProbtDiff	*** non-par ProbtDiff
**Control**	1	d4	0.84	0.00	1.87	5		
	1	d7	61.31	75.58	29.77	5		
	1	d11	79.58	61.45	36.65	5		
	1	d14	86.49	75.58	44.03	5		
	1	d18	149.10	84.55	108.25	5		
	1	d20	230.15	164.22	172.17	5		
	1	d25	310.05	207.38	192.38	5		
	1	d28	470.22	262.43	498.58	5		
**AUR 1000**	2	d4	0.84	0.00	1.87	5	1	1
	2	d7	33.52	25.40	28.61	5	0.240682	0.324151
	2	d11	54.93	34.70	40.32	5	0.587529	0.577561
	2	d14	55.97	63.35	45.02	5	0.475784	0.526316
	2	d18	66.36	59.58	64.84	5	0.161066	0.193566
	2	d20	127.36	130.60	121.80	5	0.38863	0.323199
	2	d25	205.11	179.15	143.98	5	0.512224	0.769835
	2	d28	329.83	298.64	295.24	5	0.86056	0.953777
**ATRA 10**	3	d4	1.63	0.00	3.65	5	0.917217	0.997935
	3	d7	14.83	14.10	18.17	5	0.028719	0.029321
	3	d11	27.14	27.54	30.31	5	0.090755	0.069472
	3	d14	27.84	16.32	36.18	5	0.0728	0.073694
	3	d18	20.24	23.37	20.85	5	0.020062	0.007925
	3	d20	42.41	38.96	37.93	4	0.071625	0.041401
	3	d25	97.49	121.59	66.24	4	0.09269	0.069708
	3	d28	242.02	102.22	353.58	4	0.650798	0.649378
**AUR+ ATRA10**	4	d4	0.84	0.00	1.87	5	1	1
	4	d7	20.78	16.32	24.15	5	0.05918	0.081976
	4	d11	24.37	9.18	37.53	5	0.072444	0.040439
	4	d14	33.05	24.37	26.72	5	0.108048	0.17103
	4	d18	40.17	30.98	38.56	5	0.051321	0.066363
	4	d20	47.32	20.53	66.37	5	0.061652	0.021823
	4	d25	74.94	47.59	105.28	5	0.044058	0.042833
	4	d28	75.75	16.32	140.92	5	0.214195	0.118463

*Group identification

	AUR	ATRA
1	no	no
2	yes	no
3	no	yes
4	yes	yes

**parametric statistics
***non-parametric statistics

*All p-values are comparisons to the control group (1)
light blue: marginally significant p-value
dark blue: significant p-value

Supplemental Table 8

Body weight (BW) statistics for AUR ± ATRA xenograft study*

treatment group	group	days on study	BW (g) mean	BW (g) median	SD	N	** paramet ProbtDiff	*** non-par ProbtDiff
Control	1	d-6	19.2	20.0	1.1	5		
	1	d-3	19.0	19.0	1.0	5		
	1	d-1	20.0	20.0	1.4	5		
	1	d1	18.2	18.0	1.1	5		
	1	d4	19.4	20.0	1.9	5		
	1	d7	19.8	20.0	1.8	5		
	1	d11	20.4	20.0	1.8	5		
	1	d14	21.0	20.0	1.4	5		
	1	d18	20.6	20.0	1.9	5		
	1	d20	22.0	22.0	2.0	5		
	1	d22	21.0	21.0	1.6	5		
	1	d25	20.4	20.0	1.8	5		
	1	d27	20.6	20.0	2.2	5		
	1	d28	20.9	20.6	1.7	5		
AUR 1000	2	d-6	20.0	20.0	1.4	5	0.624008	0.6654257
	2	d-3	20.0	20.0	1.4	5	0.528509	0.3701595
	2	d-1	20.0	20.0	1.4	5	1	1
	2	d1	19.8	20.0	1.1	5	0.170332	0.0949854
	2	d4	18.4	19.0	1.5	5	0.641159	0.8056753
	2	d7	18.8	20.0	1.8	5	0.606426	0.7460508
	2	d11	19.4	20.0	2.2	5	0.6567	0.7640607
	2	d14	19.9	20.0	1.6	5	0.511361	0.5059021
	2	d18	20.8	20.0	1.1	5	0.994954	0.9661383
	2	d20	20.4	20.0	1.1	5	0.385338	0.4983953
	2	d22	20.2	20.0	1.1	5	0.846334	0.8565808
	2	d25	20.0	20.0	1.4	5	0.970107	0.9730265
	2	d27	20.4	20.0	0.9	5	0.997533	0.9994252
	2	d28	20.6	20.4	0.9	5	0.975875	1
ATRA 10	3	d-6	18.4	18.0	0.9	5	0.624008	0.5813258
	3	d-3	20.8	20.0	1.8	5	0.127394	0.114954
	3	d-1	21.4	21.0	1.5	5	0.295928	0.3024975
	3	d1	20.2	20.0	1.9	5	0.071285	0.079557
	3	d4	19.4	19.0	1.7	5	1	0.9999865
	3	d7	18.4	18.0	1.7	5	0.356424	0.4415026
	3	d11	17.4	18.0	1.3	5	0.027182	0.0230053
	3	d14	17.8	18.0	1.8	5	0.008646	0.0094279
	3	d18	17.2	18.0	2.3	5	0.013575	0.0103772
	3	d20	17.0	17.0	2.6	4	0.002278	0.0027022
	3	d22	17.5	16.5	3.3	4	0.038159	0.0370921
	3	d25	17.8	18.0	2.2	4	0.102768	0.0982189
	3	d27	16.3	15.0	3.3	4	0.019647	0.1603911
	3	d28	18.8	19.7	2.2	4	0.17078	0.1470911
AUR+	4	d-6	20.2	20.0	1.5	5	0.46205	0.461187

**ATRA10**	4	d-3	18.2	18.0	1.1	5	0.681055	0.5386893
	4	d-1	19.0	19.0	1.2	5	0.547833	0.5041575
	4	d1	18.6	18.0	0.9	5	0.930983	0.8775002
	4	d4	18.4	18.0	1.1	5	0.641159	0.6893583
	4	d7	18.0	18.0	0.0	5	0.185274	0.178759
	4	d11	18.6	18.0	0.9	5	0.232076	0.1904138
	4	d14	18.6	18.0	0.9	5	0.049687	0.0297687
	4	d18	17.6	18.0	0.9	5	0.029442	0.0095328
	4	d20	18.2	18.0	1.3	5	0.011544	0.0049225
	4	d22	18.0	18.0	1.2	5	0.061637	0.0223469
	4	d25	17.6	18.0	1.7	5	0.062523	0.0535349
	4	d27	19.2	20.0	1.6	5	0.602709	0.6892826
	4	d28	18.0	17.8	1.7	5	0.028941	0.0216576

*Group identification

	AUR	ATRA
1	no	no
2	yes	no
3	no	yes
4	yes	yes

**parametric statistics
***non-parametric statistics

*All p-values are comparisons to the control group (1)
light blue: marginally significant p-value
dark blue: significant p-value
